# Gorham’s disease: treatment with an autologous iliac bone graft and a reverse total shoulder arthroplasty

**DOI:** 10.1186/s12891-019-2436-0

**Published:** 2019-02-13

**Authors:** Linjie Feng, Yaoping Wu, Xiangqian Yu, Wenguo Zhao

**Affiliations:** 1 The Department of Orthopedics, North China University of Science and Technology Affiliated Hospital, No.73 Jianshe South Road, Tangshan, Hebei 063000 People’s Republic of China; 2 The Department of Orthopedics, Xijing Hospital, Military Medical University of Air Force (The Fourth Military Medical University), Xi’an, Shanxi 710032 People’s Republic of China

**Keywords:** Gorham’s disease, Autologous iliac bone graft, Reverse total shoulder arthroplasty, Shoulder joint function

## Abstract

**Background:**

Gorham’s disease (GSD) is a rare osteolytic disease with unclear etiology, and no known prevention or effective treatment. Here we report a new surgical treatment for a case of GSD in September 2017.

**Case presentation:**

We report GSD in a 52-year-old woman. She had disappearance of her humeral head and a defect of the glenoid bone in her left shoulder joint, which were serious obstacles to joint function. We used an autologous iliac bone graft to repair the glenoid bone defect and a reverse total shoulder arthroplasty. After surgery, humeral osteolysis did not continue, and her shoulder function recovered well.

**Conclusions:**

This case suggests that autologous bone grafting can still be used to treat GSD despite it being an osteolytic disease. The successful treatment suggests that this method could be used for GSD in other bones.

## Background

Gorham’s disease (GSD) was first reported by Jackson in 1838 then later in 1872 [1, 2]. In 1955, Gorham and Stout defined a specific disease entity from a review of 24 cases in the literature [3]. GSD can affect any bone in the body, but it has a predilection for bones of the shoulder and pelvic girdle [4–7]. It presents as progressive idiopathic osteolysis of one bone or contiguous bones around one focus, without respect for joint boundaries [3–6], and it may arrest spontaneously. Several methods have been reported to deal with this disease in the literature, such as radiotherapy, pharmaceuticals, complete resection, or a custom-made prosthesis to replace the resected bone and joint [8–14].

We report a case of GSD with extremely rapid progression of idiopathic osteolysis in the proximal humerus and glenoid in only 2 months [15]. It was treated with a reverse total shoulder arthroplasty with simultaneous reconstruction of the glenoid bone defect using autologous iliac bone grafts. The shoulder joint function recovered well 6 months after surgery.

## Case presentation

**Fig. 1:**
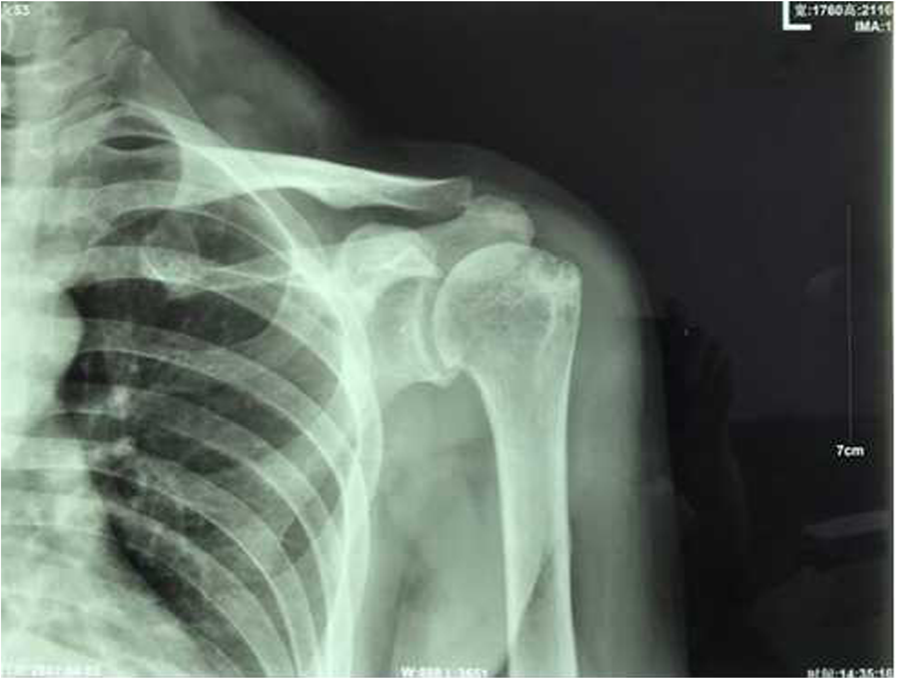
Complete bone structure of the shoulder joint with no obvious bone destruction

**Fig. 2:**
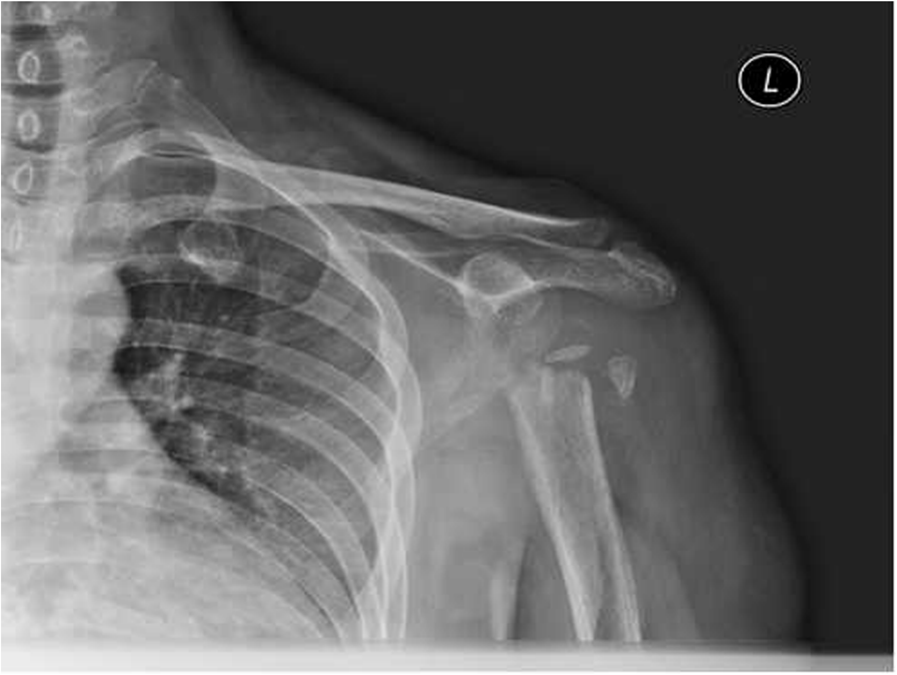
Extensive destruction of the proximal humerus with glenoid bone defect and eventual complete destruction of the humeral head

**Fig. 3:**
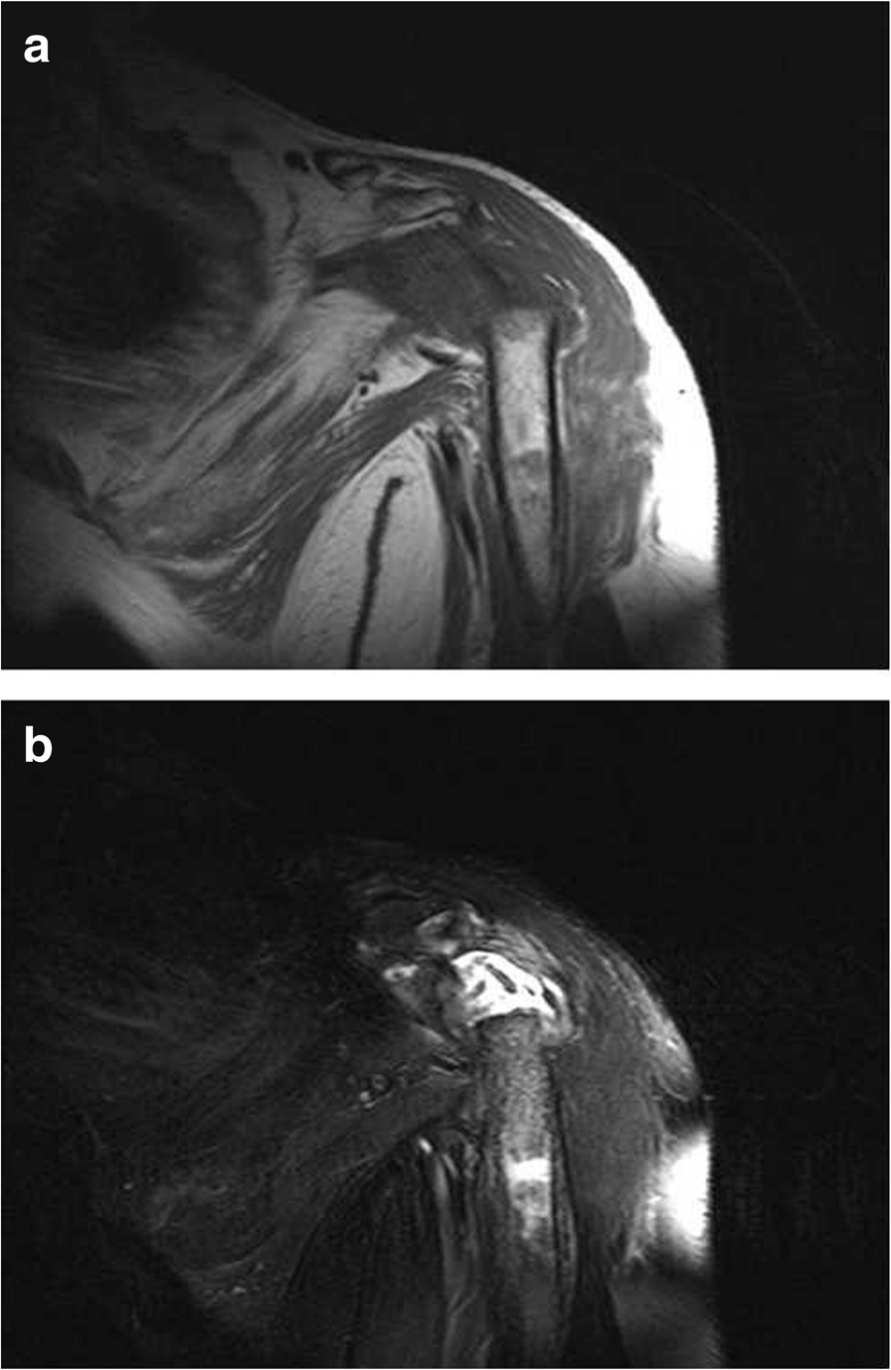
Magnetic resonance imaging. **a** Hypointense signal on T1-weighted images. b Areas of isointensity and hyperintensity on the T2-weighted image

A 52-year-old woman presented with pain and active function loss in her left shoulder, and was admitted to our hospital in June 2017. Her shoulder problem had started 3 months earlier, and there was no history of trauma or fracture. At first the pain was intermittent and bearable, but then gradually increased. On examination, there were no positive signs except for localized pain. A radiologic examination on 3 April 2017 found no destruction of the shoulder (Fig. 1). Pain at the shoulder joint became gradually aggravated, together with the appearance of shoulder joint dysfunction. Two months later, physical examination revealed mild swelling of the shoulder, and markedly restricted shoulder and elbow motion. Mild distal nerve function defects appeared gradually. Radiography on 3 June 2017 showed that the head of the humerus had disappeared within the past 2 months (Fig. 2), which was confirmed by magnetic resonance imaging (Fig. 3a, b).

**Fig. 4:**
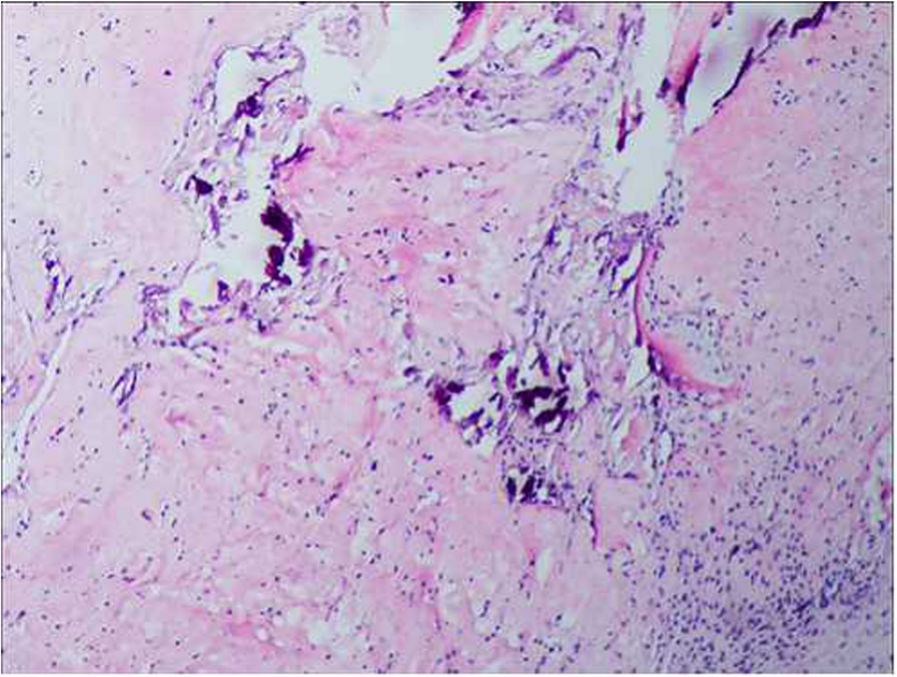
Fibrous connective tissue replacing bony tissue. Small areas of bony trabeculae with occasional osteoclasts were evident

**Fig. 5:**
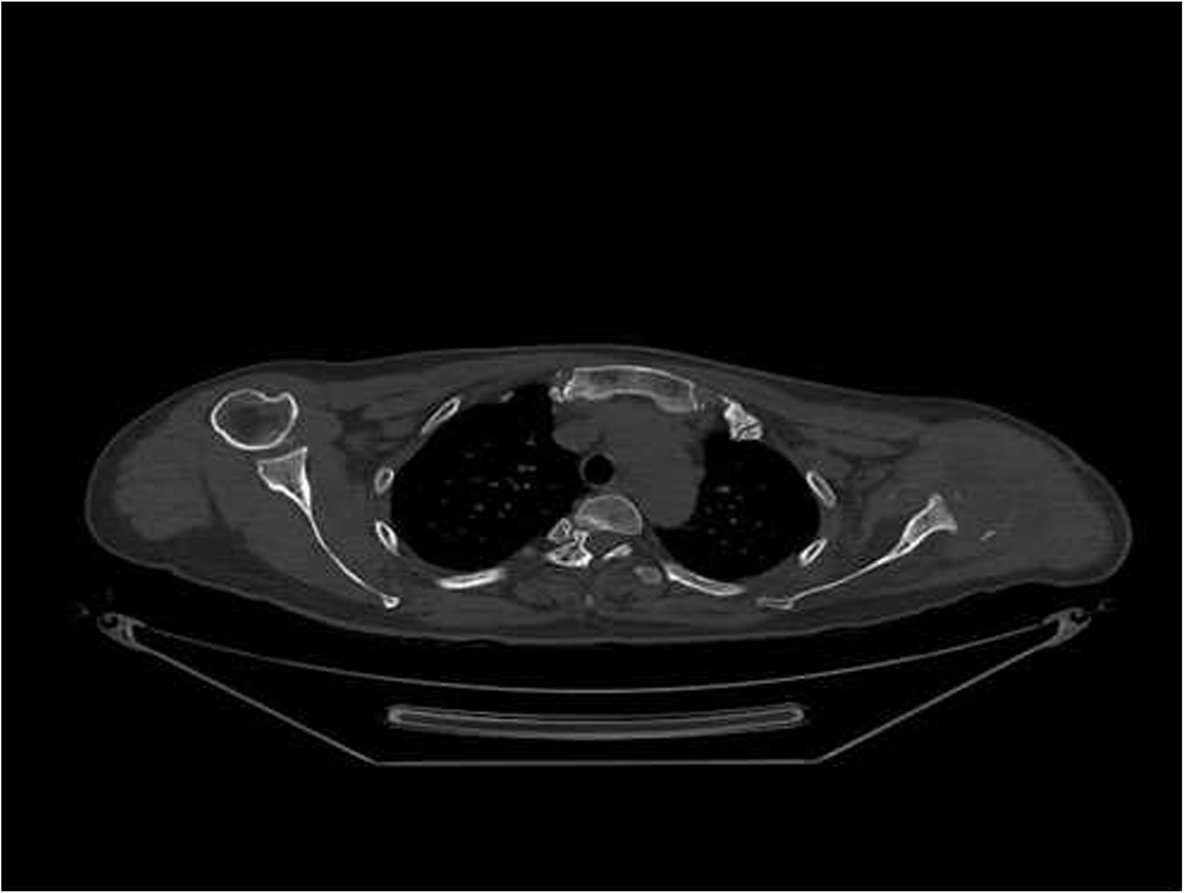
Computed tomography scan of the shoulder (coronal slice)

The patient is a healthy, active individual with no history of weight loss, anorexia, or fever during this period. General and systemic examination findings were within normal limits. Routine laboratory investigations were also normal, including levels of serum calcium, phosphate, alkaline phosphatase, high-sensitivity C-reactive protein, and erythrocyte sedimentation rate. An open biopsy of the lesion revealed that the bony tissue had been replaced by fibrous connective tissue, and small areas of bony trabeculae with occasional osteoclasts were visible (Fig. 4). There was no evidence of malignancy or tuberculosis. Because of the lack of any clinical findings or supporting data for other causes, the features were confirmatory of GSD. Computed tomography of the shoulder joint (Fig. 5) revealed a bony defect of the glenoid cavity.

**Fig. 6:**
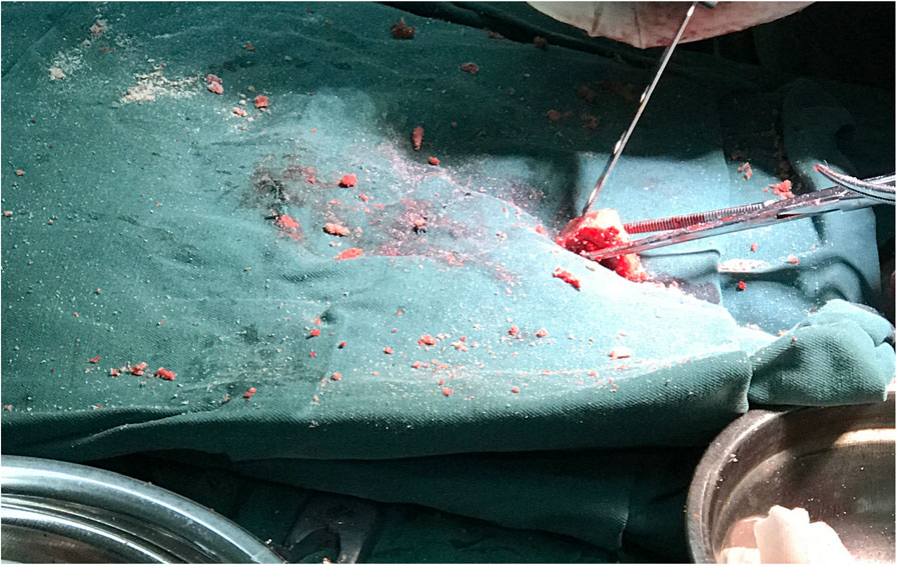
Repair of the autologous iliac bone

**Fig. 7:**
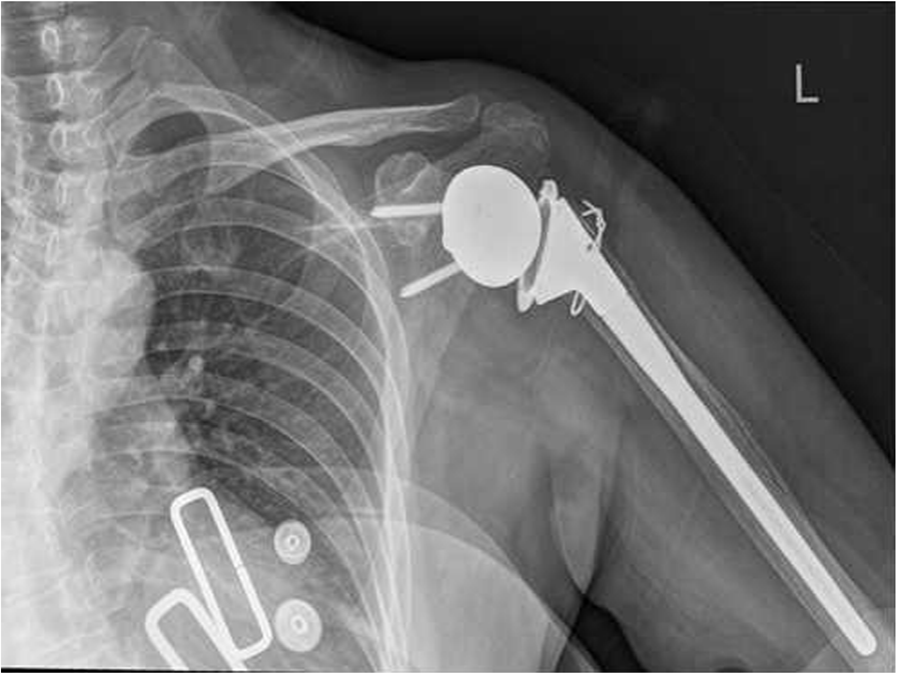
X-ray of the shoulder after the reverse total shoulder arthroplasty

**Fig. 8:**
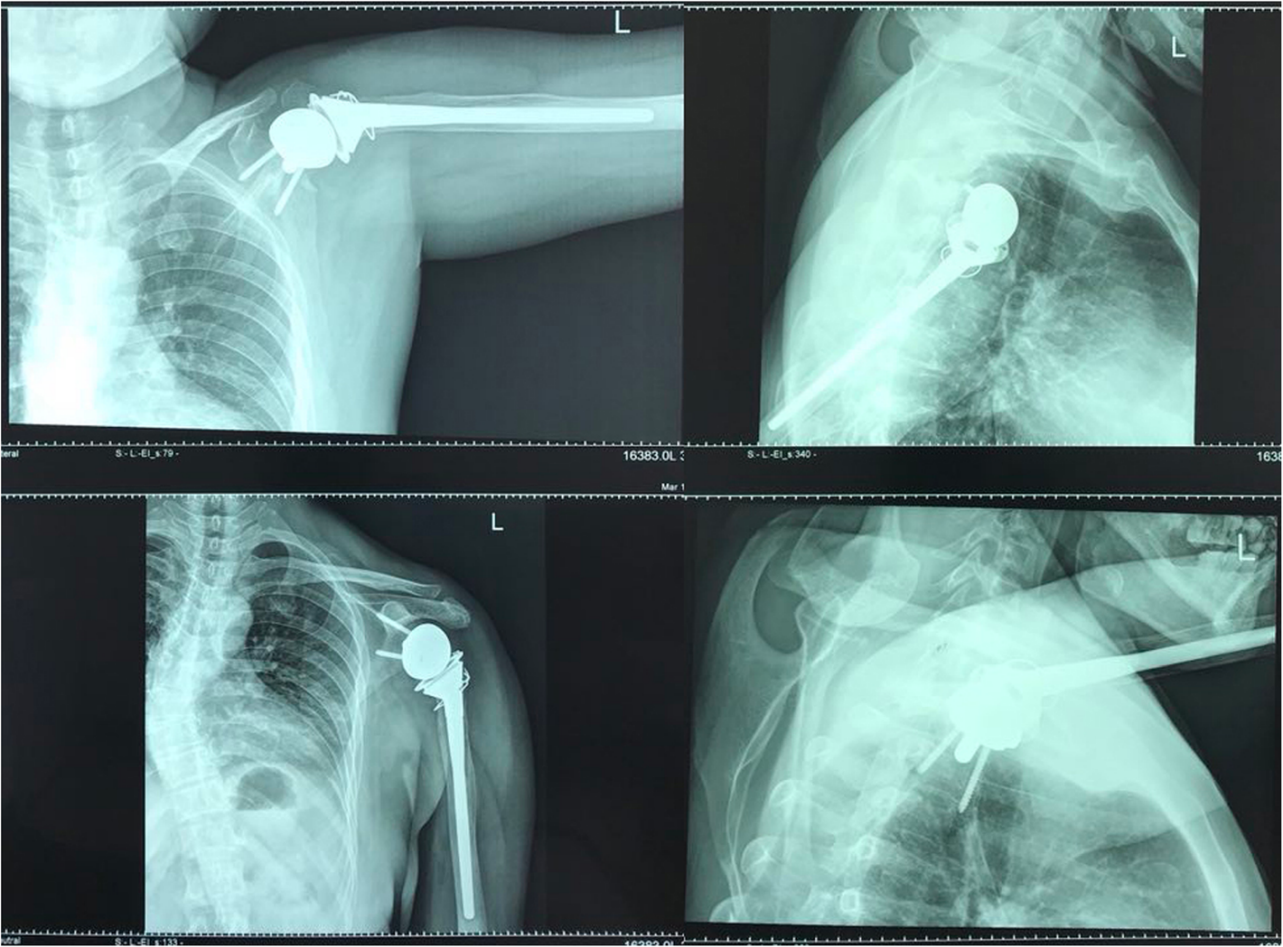
X-ray of the shoulder 3 months after surgery

**Fig. 9:**
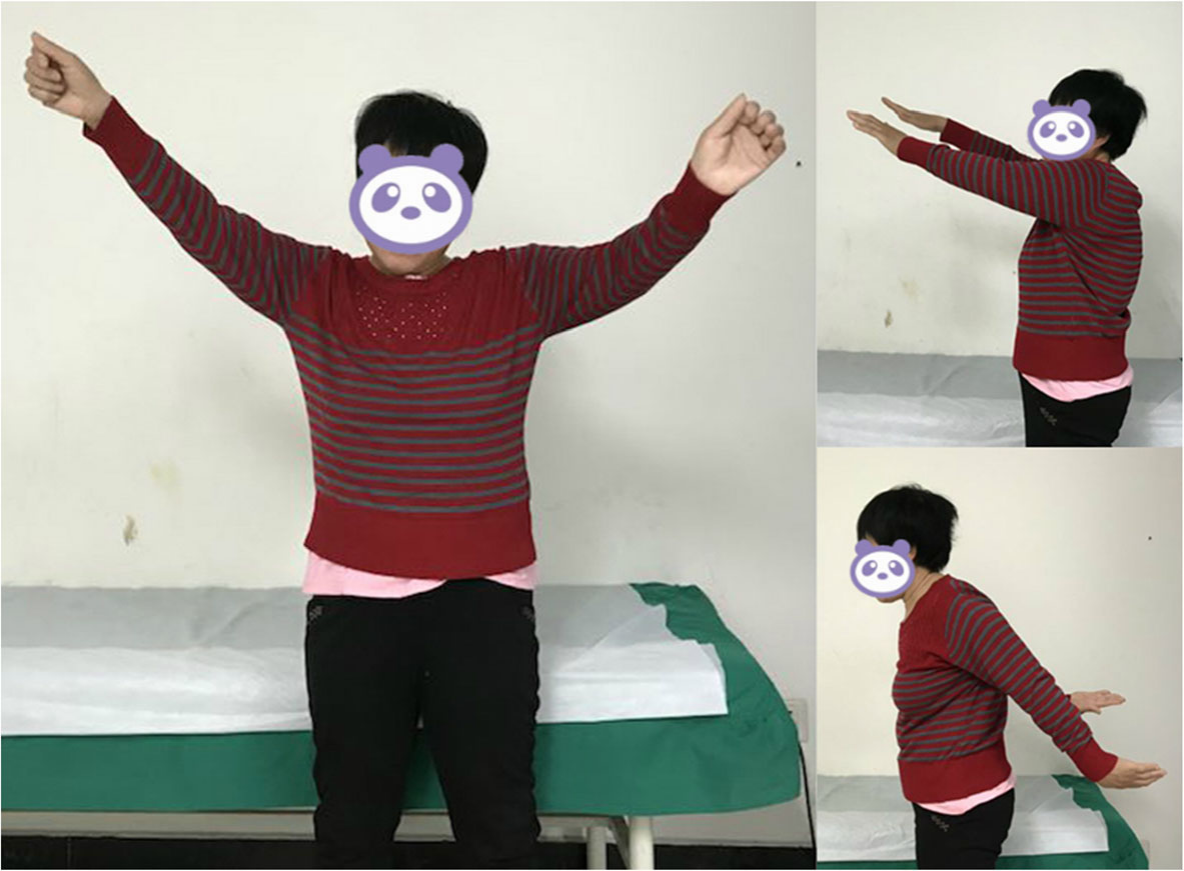
Active range of movement 3 months after surgery

We performed surgery involving glenoid cavity amplification with an autologous iliac bone graft and a reverse total shoulder arthroplasty. The grafts were from autologous iliac bone (Fig. 6, Fig. 7). A deltopectoral approach was used on the shoulder, and necrotic and dissolving bone tissue was removed. Reconstruction of the glenoid was carried out with autologous iliac bone and installation of reverse shoulder prosthesis. Postoperatively, the arm was placed in a sling for 3 months. Passive elevation and external rotation were allowed 2 weeks after the operation. Three months later, sling use was discontinued, and active range of movement was initiated. Six months after surgery, the patient is pain-free with more than 90° of active abduction, 100° of forward flexion, and 30° of shoulder posterior extension. She also has good functional use of her shoulder (Fig. 8, Fig. 9).

## Discussion and conclusions

GSD is an unusual nonmalignant condition characterized by spontaneous and massive osteolysis. More than 300 cases of GSD have been described in the literature, but the underlying cause remains unknown [8, 9].

Various strategies are used to treat GSD [9–11], but in the present case it was important to preserve the function and shape of the shoulder joint. In particular, the problem of osteolysis had to be overcome, and the defect of the glenoid bone using autologous or allogeneic bone had to be considered. Surgery is a suitable treatment to reduce or stop the progression of GSD, but conservative treatment could result in loss of shoulder function or even amputation. In this case, the patient had extensive and rapid involvement of the glenoid and proximal humerus. We chose replacement prosthesis of the shoulder rather than amputation to preserve the function of the shoulder joint. Fortunately, the operation not only retained this function but also stopped the osteolysis damage. Up to now, the autologous bone graft was integrated with the shoulder glenoid and prosthesis, and no prosthesis loosening occurred. The patient was very satisfied with the treatment outcome.

Using an autologous bone graft for the reconstruction of the shoulder glenoid was a critical step in the present case that provided a basis for the installation of the shoulder prosthesis. This method of operation has important clinical significance and provides an operative reference for the treatment of GSD or similar conditions. The cause of GSD, duration of osteolysis, and the identification of factors that prevent or slow down development of the disease require further study.

## Abbreviation

GSD: Gorham’s disease
